# A gene expression-based risk model reveals prognosis of gastric cancer

**DOI:** 10.7717/peerj.4204

**Published:** 2018-01-02

**Authors:** Xiaorong Deng, Qun Xiao, Feng Liu, Cihua Zheng

**Affiliations:** 1 Department of Gastrointestinal Surgery, The Second Affiliated Hospital of Nanchang University, Nanchang, Jiangxi, China; 2 Department of Hepatobiliary Surgery, The First Affiliated Hospital of Hunan College of Traditional Chinese Medicine, Zhuzhou, Hunan, China; 3 Department of General Surgery, The First Affiliated Hospital of Nanchang University, Nanchang, Jiangxi, China; 4 Jiangxi Medical College of Nanchang University, Nanchang, Jiangxi, China

**Keywords:** Gatric cancer, Prognosis, Model

## Abstract

**Background:**

The prognosis of gastric cancer is difficult to determine, although clinical indicators provide valuable evidence.

**Methods:**

In this study, using screened biomarkers of gastric cancer in combination with random forest variable hunting and multivariable Cox regression, a risk score model was developed to predict the survival of gastric cancer. Survival difference between high/low-risk groups were compared. The relationship between risk score and other clinicopathological indicators was evaluated. Gene set enrichment analysis (GSEA) was used to identify pathways associated with risk scores.

**Results:**

The patients with high risk scores (median overall survival: 20.2 months, 95% CI [16.9–26.0] months) tend to exhibit early events compared with those with low risk scores (median survival: 70.0 months, 95% CI [46.9–101] months, *p* = 1.80e–5). Further validation was implemented in another three independent datasets (GSE15459, GSE26253, GSE62254). Correlation analyses between clinical observations and risk scores were performed, and the results indicated that the risk score was not significantly associated with gender, age and primary tumor size but was significantly associated with grade and tumor stage. In addition, the risk score was also not influenced by radiation therapy. Cox multivariate regression and three-year survival nomogram suggest that the risk score is an important indicator of gastric cancer prognosis. GSEA was used to identified KEGG pathways significantly associated with risk score, and signaling pathways involved in focal adhesion and the TGF-beta signaling pathway were identified.

**Conclusion:**

The risk score model successfully predicted the survival of 1,294 gastric cancer samples from four independent datasets and is among the most important indicators in clinical clinicopathological information for the prognosis of gastric cancer. To our knowledge, it is the first report to predict the survival of gastric cancer using optimized expression panel.

## Introduction

Gastric cancer is among the most lethal of cancers worldwide. According to most recent statistical reports in 2015, in China, 679,100 new cases and 498,000 deaths were estimated (Chen et al., 2016). Clinical indicators, including TNM staging, were proven to be effective indicators of prognosis (Wittekind, 2015). Additionally, the molecular classification also plays a powerful role in prognosis (Chen, Xu & Zhou, 2016). However, the classification effect of the staging system is still unfavorable. Thus, molecular biomarkers were needed to predict the survival of gastric cancer patients.

In recent decades, molecular biomarkers for gastric prognosis have been widely reported (Arigami et al., 2013; Guo et al., 2013; Liu et al., 2015; Rachidi et al., 2013). PD-L1 and MET1 co-expression predicted a poor survival of gastric cancer, with shorter overall survival rate and disease-free survival rate (Kwon et al., 2017). The low expression of BUB1 also suggested a poor prognosis (Stahl et al., 2017), and EPHB4 showed a similar pattern for prognosis (Yin et al., 2017). In addition, lncRNAs including SNHG and PCAT-1 were also reported to be associated with the proliferation, migration and prognosis of gastric cancer (Cui, Wu & Qu, 2017; Hu et al., 2017). However, single molecular biomarkers often fail to predict the survival of gastric cancer due to their heterogeneity, while transcriptome-based classification includes redundant information. However, the multiple molecular biomarker-based model has been proven to be robust across datasets and has been implemented in cancer (Bou Samra et al., 2014; Chang et al., 2014; Kim et al., 2014; Salazar et al., 2011; Wu et al., 2012).

In this work, genes significantly associated with survival were identified. Using these genes, the machining learning (random forest) method and Cox regression, a risk score model was developed. The risk score successfully divided the patients with good and poor prognosis. The robustness of the model was further validated in another three independent datasets. Clinical correlation analysis has shown that the score is not associated with other clinical information but was significantly correlated with primary tumor stage. Additionally, the score was effective for patients who underwent radiotherapy or not. KEGG pathway analysis showed that various cancer-related signaling pathways and focal adhesion pathways were significantly enriched.

## Material and Methods

### Data manipulation

The raw microarray data files were downloaded as GEO (https://www.ncbi.nlm.nih.gov/geo) according to the provided accession numbers. After pre-processing, including background correction and Robust Multichip Average (RMA) normalization using the R package “affy”, probes in each dataset and platform were matched to HUGO gene symbols using the manufacturer’s provided annotation files. If a single gene matched multiple probes, the average value of the probes was calculated as the relative expression of the corresponding gene. Clinical observations, including survival information, were downloaded from the same website along with the raw data. The TCGA dataset was downloaded from the UCSC Xena website (https://tcga.xenahubs.net/) and further converted to log 2 transformed RPKM values according to the website’s provided protocol. Clinical information was also downloaded via UCSC Xena. To normalize the data among batches and platforms, *z*-scores were calculated for each patient in each dataset.

### Gene selection and model development

Correlation analyses between overall survival and the relative expression value of each gene were evaluated with Cox univariate regression with function “coxph” in the R package “survival”, and genes significantly associated with overall survival (*p* < 0.01) in both TCGA and GSE15459 were retained for further analysis. Genes not significantly different in normal and tumor tissues (*p* > 0.05) in the TCGA dataset were excluded. Afterwards, random forest variable hunting was used to optimize the panel content to develop the prediction model. After 100 repeats and 100 iterations, thirteen genes were selected. Based on the relative expression of these genes, a Cox multivariate model was carried out to develop the risk score model, and the risk score formula was calculated as follows:

\begin{eqnarray*}\text{Risk score}=\sum _{i}^{n}{x}_{i}\ast {\beta }_{i} \end{eqnarray*}

where *x*_*i*_ indicates the *z*-score transformed relative expression level of gene *i*, and *β*_*i*_ refers to coefficient of gene *i*.

### Statistical analysis and Gene Set Enrichment Analysis (GSEA)

The Cox multivariate and univariate regression was carried out with the R package “survival”, and random forest variable hunting was implemented using the R package “randomForestSRC” (Ishwaran et al., 2014) with 100 repeats and 100 iterations. The clinical correlation between risk score and clinical observations was calculated with Student’s *t*-test. The nomogram for the one-year survival rate was calculated using the R package “rms”. Three-year survival ROC was plotted using R functions in the package “pROC” (Robin et al., 2011). GSEA analysis was implemented based on TCGA dataset using Gene Set Enrichment Analysis (Subramanian et al., 2005) (GSEA) java software. Differential gene identification in the TCGA dataset was implemented with the R package “limma” using log 2 transformed RPKM values.

## Results

### Gene selection and model development

**Figure 1:**
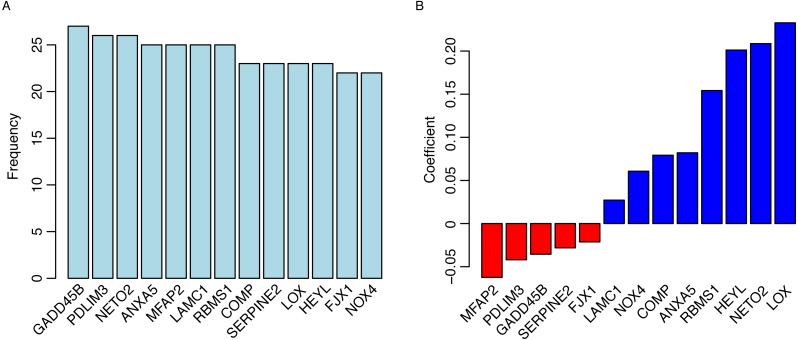
Gene selection and model development. The frequency of genes presented in random forest variable hunting (A) and the coefficient for each gene (B).

To identify the survival-related genes, Cox univariate regression between overall survival and gene expression was implemented in both the TCGA dataset (*N* = 380) and GSE15459 datasets to remove the false discovery. Genes significantly associated with overall survival (*p* < 0.01) in both datasets were considered survival-related genes (termed gene list 1). Differential genes between normal and tumor tissues were identified, and the expression levels of genes that were not significantly different between normal and tumor tissues were excluded from gene list 1 (termed gene list 2). Considering that redundant information exists in these genes and excessive genes may hinder the utilization of the model, a machine learning method called random forest variable hunting was employed to reduce the complexity and optimize the gene combination. Thirteen genes were selected for further analysis (Fig. 1A, Table 1). The risk score was calculated as follows: Riskscore = (0.060675302∗NOX4) + (−0.021259171∗FJX1) + (0.20119841∗HEY L) + (0.23276666∗LOX) + (−0.028145979∗SERPINE2) + (0.079260655∗COMP) + (0.154255568∗RBMS1) + (0.027185616∗LAMC1) + (−0.062461521∗MFAP2) + (0.082089956∗ANXA5) + (0.208657253∗NETO2) + (−0.041982925∗PDLIM3) + (−0.035559668∗GADD45B), where the gene symbol represents the gene expression values.

**Table 1:** Parameters of variables. Hazard ratio, 95% confidence interval, and *p* values of candidate genes according to Cox univariate and multivariate regression.

	Univariate Cox regression			Multivariate Cox regression		
	HR	95% CI	*p*value	HR	95% CI	*p*value
NOX4	1.3	1.1–1.5	0.00312	0.96	0.71–1.28	0.76338
FJX1	1.2	1.1–1.5	0.00854	0.96	0.78–1.19	0.71577
HEYL	1.4	1.2–1.6	0.00028	1.3	0.99–1.71	0.05764
LOX	1.3	1.1–1.5	0.0015	1.1	0.86–1.42	0.44066
SERPINE2	1.2	1.1–1.5	0.00701	0.91	0.73–1.14	0.42652
COMP	1.2	1.1–1.4	0.00696	1.08	0.84–1.38	0.53894
RBMS1	1.3	1.1–1.6	0.00147	1.16	0.91–1.48	0.21607
LAMC1	1.3	1.1–1.5	0.00175	1.07	0.86–1.34	0.53129
MFAP2	1.3	1.1–1.5	0.00222	0.96	0.72–1.26	0.75653
ANXA5	1.3	1.1–1.5	0.00456	1.18	0.95–1.46	0.13056
NETO2	1.3	1.1–1.5	0.00697	1.35	1.11–1.64	0.00245
PDLIM3	1.2	1.1–1.4	0.005	0.96	0.74–1.25	0.78218
GADD45B	1.3	1.1–1.5	0.00428	1.06	0.85–1.33	0.59772

The coefficients of genes are shown in Fig. 1B. High expression of genes with positive coefficients positively correlated the risk score value, thus, these genes are tumor genes. While high expression of genes with negative coefficients negatively correlated the risk score value, thus these genes are tumor suppressor genes.

**Figure 2:**
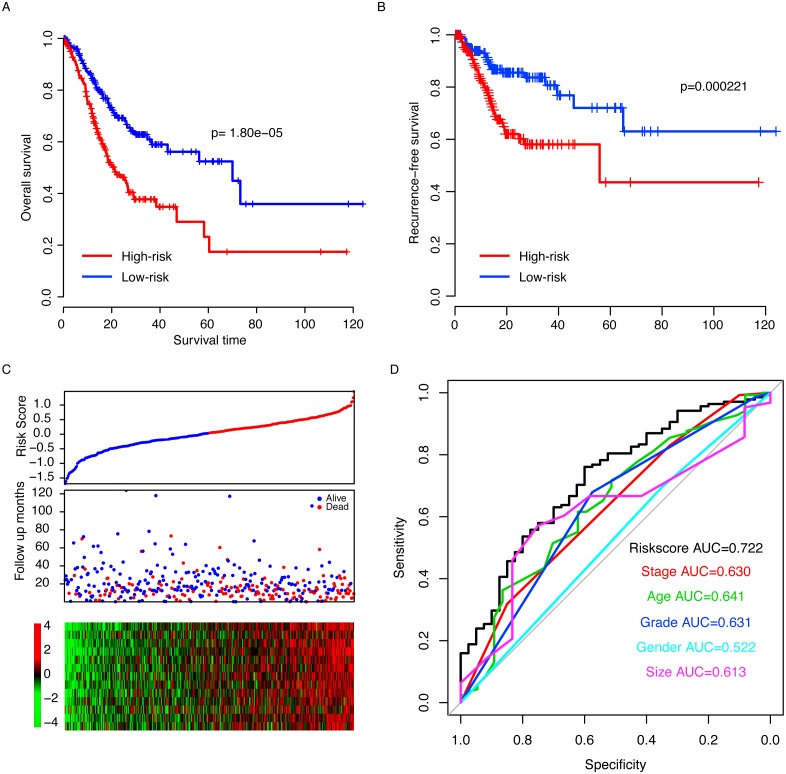
Risk score in the TCGA dataset. The high-risk group had a significantly longer overall survival (OS) time than low risk group (A), and a similar pattern was observed for recurrence-free survival (RFS, B). The detailed survival information of samples, risk score and gene expression (C) and three-year survival ROC were also calculated (D).

**Figure 3:**
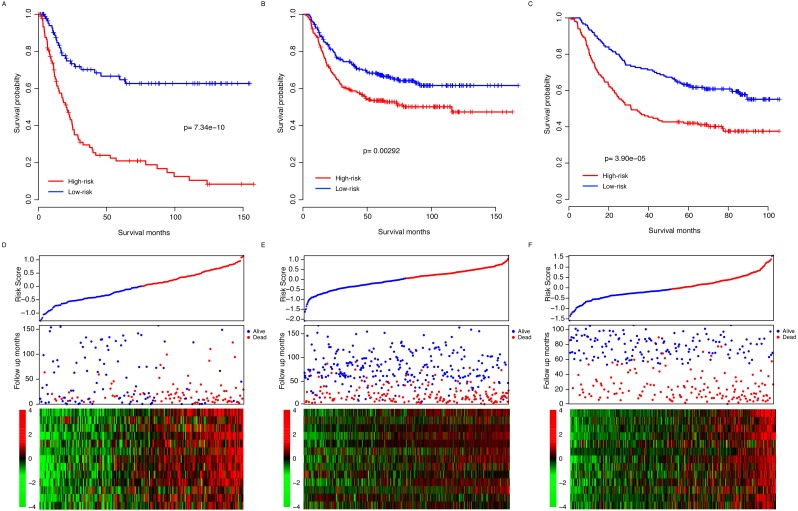
Risk score performance validation. The performance of risk in predicting survival was validated in the GSE15459 (A), GSE26253 (B) and GSE62254 (B) datasets. The detailed survival information and gene expression of the three datasets (D–F) also resembled the profile of the training dataset (TCGA).

### Risk score predicts the survival of the TCGA dataset

The performance of the risk score was evaluated in the training datasets by dividing the samples in the TCGA dataset into two subgroups, high-risk and low-risk, using the median risk score as a cutoff (0.00436). The survival time of the low risk score is 70.0 (95% CI [46.9–101]) months, which is significantly longer (*p* = 1.80e–5, Fig. 2A) than the high-risk group (20.2 months, 95% CI [16.9–26.7]). The recurrence-free survival (RFS) was also compared between the two groups, and the RFS of the low-risk group is also significantly longer than the high-risk group (*p* = 0.000221, Fig. 2B). As shown in Fig. 2C, along with an increase of risk score, patients tend to exhibit early events, a high expression of oncogenes and a low expression of tumor repressor genes. The three-year survival area under the receiving operating characteristic (AUROC) curve was calculated, and the AUROCs of risk score, stage, age, grade, gender and primary tumor size were 0.722, 0.630, 0,641, 0.631, 0.522 and 0.613 (Fig. 2D), suggesting that risk score is an important indicator of the survival of gastric patients.

**Figure 4:**
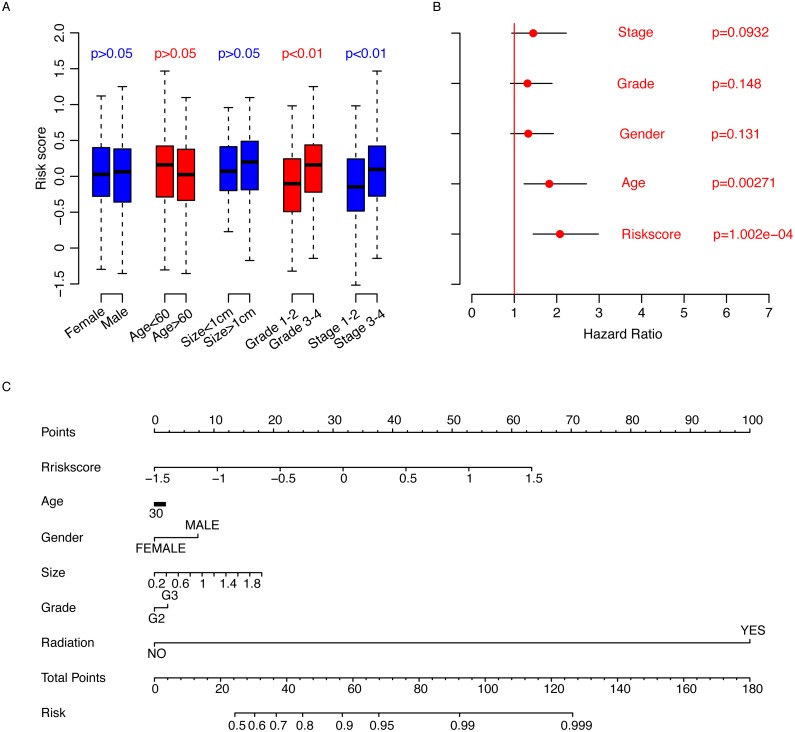
Clinical observations and risk score. The risk score is not associated with other clinical observations (A) besides stage and grade, and it is an important indicator of survival (B) according to Cox multivariate regression. The three-year survival nomogram was plotted to facilitate the utilization of the risk score (C).

### Risk score performance validation

The observed prognostic performance of the risk score in the training dataset (TCGA) may have resulted from over-fitness between the data and model. To test the robustness of the model, after locking the coefficient of each gene, the risk score of each sample in each dataset was evaluated. The validation datasets include another three independent datasets, GSE15459 (*N* = 192), GSE26253 (*N* = 422) and GSE62254 (*N* = 300). By dividing the patients of each dataset into high-risk and low-risk groups according to the median risk score as the cutoff in each dataset, the survival difference of these two subgroups was evaluated. The survival time in the high-risk group was significantly shorter than the low-risk group in all three datasets (*p* = 7.34e–10, 0.00292 and 3.90e–5 for GSE15459, GSE26253 and GSE62254, respectively, Figs. 3A–3C). Similar to the training dataset, along with the increase in the risk score, early death was detected in patients with a high risk score in each sample (Figs. 3D–3F). In addition, the gene expression patterns in these three datasets of these thirteen genes also resembles those in the training dataset (Figs. 3D–3F). Collectively, these results indicate that the risk score model is robust in predicting the survival of gastric patients across datasets and platforms.

### Risk score and clinicopathological information

The correlation analyses between clinicopathological information and risk score were also performed. First, we compared the risk score values in the clinical observation categories. It was noted that age (<60, >60), gender, and primary tumor size (>1 cm, <1 cm) were not significantly associated with risk score (Fig. 4A), while the risk score was significantly associated with higher grade and stage (*p* < 0.01). Subsequently, Cox multivariate regression was implemented to evaluate the significance of age, gender, stage, grade and risk score (Fig. 4B). The results showed that the risk score is one of the most important clinical indicators of prognosis. To facilitate the utilization of the risk score, a nomogram for three-year overall survival using the aforementioned clinical information was plotted (Fig. 4C). All these results indicate that the risk score is an important clinical indicator of gastric cancer prognosis.

**Figure 5:**
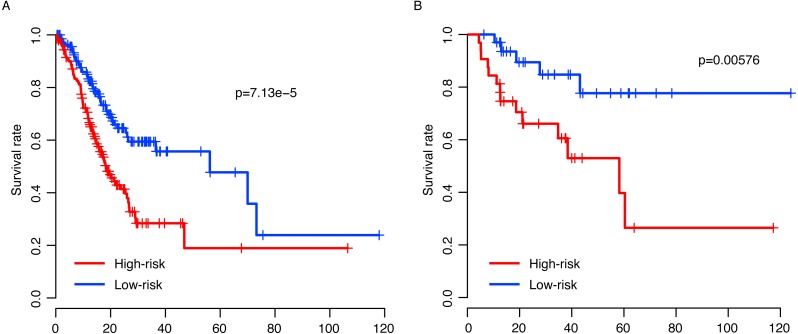
Risk score and radiotherapy. The risk score successfully predicted the survival of patients who received radiotherapy (A) or not (B).

**Figure 6:**
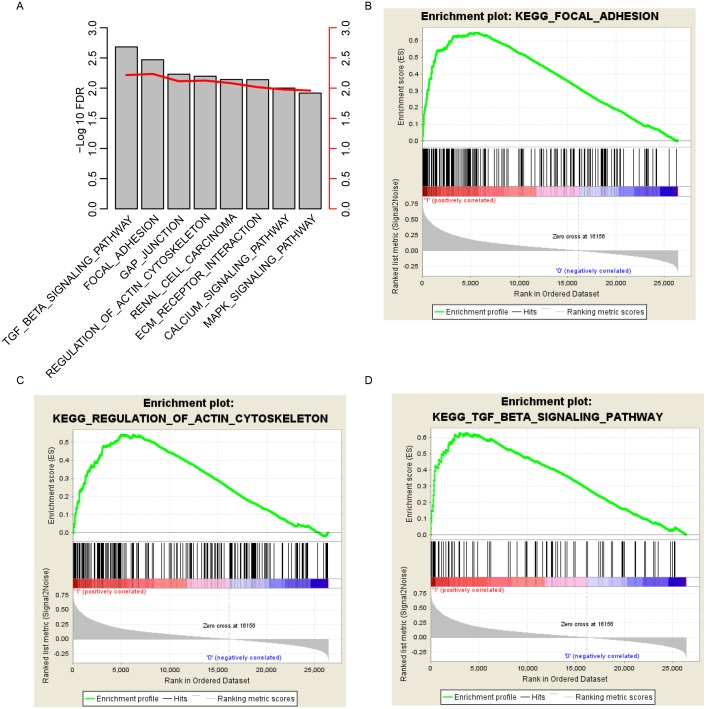
KEGG pathways associated with risk score. GSEA according to the expression of the TCGA dataset revealed a significant pathway associated with risk score (A), including focal adhesion (B), the regulation of the actin cytoskeleton (C) and the TGF-beta signaling pathway (D).

### Risk score and radiation

Radiation is among the most important adjuvant therapy methods in gastric treatment. Thus, the risk score performance in patients who underwent radiation or not was investigated to test whether it was effective in these sub-categories. The patients who did not receive radiation were divided into high-risk and low-risk groups according to the median risk score value in these samples. As expected, patients who did not receive radiation and who had higher risk scores had significantly poorer survival than those with a low risk score (Fig. 5A, *N* = 289). The survival pattern of patients who received radiation also resembled that of those without radiation (Fig. 5B, *N* = 69). These results indicate that the risk score is robust and not effected by radiation therapy.

### KEGG pathways associated with risk score

To investigate how the risk score predicted the survival of gastric cancer, we divided the samples in TCGA datasets into high-risk and low-risk groups according to the median risk score values, as previously described. GSEA was carried out to investigate the pathways that were significantly different between the high/low risk groups. Multiple cancer-related KEGG signaling pathways, including the TGF-beta signaling pathway, focal adhesion, gap junction, regulation of actin cytoskeleton and MAPK signaling pathway, were significantly enriched (Fig. 6A; false discovery rate (FDR) < 0.01). Of these pathways, focal adhesion, the regulation of the actin cytoskeleton and the MAPK signaling pathway were noted (Figs. 6B–6D). These results suggest that the risk score reflects multiple cancer statuses of gastric cells and thus predicts the survival.

## Discussion

The prognosis of gastric cancer varies due to many reasons. First, the progression status evaluated by the clinical and pathological indicators explain the prognosis, to some extent (Wittekind, 2015). Second, the treatment method, including the surgery (R0/R1/R2) and treatment method (adjuvant therapy and targeted therapy), also influence the outcome of gastric cancer patients (Chan et al., 2016; Song et al., 2017). The third reason is the biological heterogeneity of gastric cancer, which has an important impact on carcinogenesis and development. This is the reason why biomarkers are needed for gastric cancer.

Although single biomarkers have been reported in recent years (Arigami et al., 2013; Chan et al., 2016; Guo et al., 2013; Hu et al., 2017; Liu et al., 2015; Rachidi et al., 2013; Stahl et al., 2017), the performance of a single biomarker is not robust across datasets, which results from the biological heterogeneity of gastric cancer. One gene was detected to be significantly associated with survival in all four datasets. However, the multiple gene-based model utilized the complement of genetic information and effectively removed the redundancy of the genome. Thus, the multiple gene-based model is effective in determining the prognosis of multiple cancer types (Kim et al., 2014; Massari et al., 2015; Zhang et al., 2017).

One of the most important limitations of this study is that all samples involved in this study were retrospectively obtained, and clinicopathological indicators were not available. For example, time to metastasis, molecular subtypes including HER2 status, and anatomical location were not available for most datasets. Another important limitation of this study is that the relative expression values were *z*-score transformed; thus, a pooled dataset is needed to facilitate the utilization of this model.

## Conclusion

The risk score model is robust and useful in predicting the survival of gastric cancer.

##  Supplemental Information

10.7717/peerj.4204/supp-1 Supplemental Information 1 Code for analysis The code for variable hunting and model development.

## Additional Information and Declarations

### Data Availability

The following information was supplied regarding data availability: The raw data is publicly available in GEO with accession numbers GSE15459, GSE26253 and GSE62254. The other data were downloaded from Xena (xena.ucsc.edu):
https://xenabrowser.net/datapages/?hub=https://tcga.xenahubs.net:443.
